# Assessing Relationships between Physically Demanding Work and Late-Life Disability in Italian Nonagenarian Women Living in a Rural Area

**DOI:** 10.3390/ijerph19148880

**Published:** 2022-07-21

**Authors:** Gemma Lombardi, Silvia Pancani, Francesca Lorenzini, Federica Vannetti, Guido Pasquini, Roberta Frandi, Nona Turcan, Lorenzo Razzolini, Raffaello Molino Lova, Francesca Cecchi, Claudio Macchi

**Affiliations:** 1 IRCCS Fondazione Don Carlo Gnocchi, 50143 Florence, Italy; glombardi@dongnocchi.it (G.L.); fvannetti@dongnocchi.it (F.V.); gpasquini@dongnocchi.it (G.P.); rfrandi@dongnocchi.it (R.F.); nturcan@dongnocchi.it (N.T.); rmolino@dongnocchi.it (R.M.L.); fcecchi@dongnocchi.it (F.C.); claudio.macchi@unifi.it (C.M.); 2 Medical School, University of Florence, 50134 Florence, Italy; fralor90@gmail.com; 3 Department of Neurofarba, University of Florence, 50139 Florence, Italy; dottlorenzo.razzolini@gmail.com; 4 Department of Experimental and Clinical Medicine, University of Florence, 50134 Florence, Italy

**Keywords:** nonagenarians, physically demanding work, functional limitations, aging, disability

## Abstract

As more and more persons live into their 90s and beyond, investigating causes of disability in the oldest-old population is relevant for public health implications to plan preventive strategies and rehabilitation interventions. A negative association between physically demanding work and midlife physical function has been shown, but there is a paucity of longitudinal studies investigating possible work-related long-term effects in the oldest old. This study investigates the relationship between physically demanding work exposure and late-life physical performances, disability, general health status, and quality of life in a sample of women aged 90 years and over inside the Mugello Study. Sociodemographic data, cognitive and functional status, lifestyle, medical history, drug use, and work history were collected from 236 participants. Farmers had a lower percentage of individuals with preserved independence in basic activities of daily living compared to other occupations. However, in the multivariate analysis, only a higher cognitive function remained associated with functional independence. While confirming the well-known association between cognitive and functional decline in very old age, our results do not support the hypothesis that the negative effects of physical work exposure observed in midlife are relevant to predict disability in nonagenarian women.

## 1. Introduction

As more and more persons live into their 90s and beyond [1], investigating causes of disability in the oldest-old population, people aged 85 years and above [2], is relevant for public health implications. Many studies have shown that both medical diseases and geriatric conditions are related to functional disabilities in the “young-old”, people aged between 65 and 74 years [2], whereas, in the oldest-old, impaired cognition seems to represent one of the main predictors of disability, consistently identified in different studies together with other factors such as age [3,4], presence of comorbidities [5], depression [3,5,6], lower physical activity [6], higher BMI [6], and higher medication intake [6]. As one-third of the nonagenarians, people between 90 and 99 years old, need help in at least one basic activity of daily living [4], understanding the interplay between main lifetime occupation and disability in later life is of paramount importance to plan preventive strategies and rehabilitation interventions.

Despite the paucity of long follow-up studies, there is evidence of a negative association between physical exposures throughout working life and midlife (i.e., between 55 and 64 years old [2]) physical function [7,8]. A higher risk of disability has also been associated with physically demanding work [9,10,11,12], low education, poor financial assets, low occupational status, and unfavorable health behaviors [10,13]. In the same way, results from an Italian study on a cohort of elderly persons aged seventy and over revealed that farmers, assumed as workers exerting a physically demanding job, were more disabled in performing instrumental activities of daily living, compared to white-collar workers, although this relationship was no more significant after the adjustment of the analysis for the cognitive status [14]. Moreover, a strong relationship between occupation type and general health status [15] as well as quality of life [16,17], and between occupation type and all-cause mortality [18,19,20] was found. Finally, whereas current guidelines recommend moderate to vigorous leisure-time physical activity (LTPA) to promote health, the effect of high level occupational physical activity (OPA) on comorbidities development seems to be the opposite, configuring the “physical activity paradox” [18].

Differences between healthy and detrimental physical activity habits may reflect inequalities in socioeconomic conditions, representing per se a proxy of health disparities [13]; however, epidemiological studies document that high OPA increases the risk for cardiovascular disease and mortality outcomes, even after extensive adjustments for other risk factors including socioeconomic status, LTPA, and other health behaviors [11,21]. In this regard, a recent general population study conducted on 104,046 adults in Denmark confirmed the association between higher LTPA and reduced major adverse cardiovascular events and all-cause mortality risk, and between OPA and increased risks, independent of each other [18].

Recent data suggest that caution should be used when merging results for men and women because a gender difference exists in terms of the type of stressors experienced and vulnerability due to stressors exposure [15,19,22,23,24]. Moreover, a “gender effect” on occupation type is frequent in older Mediterranean populations, where it is difficult to find either man reporting as “housemen” or women reporting as “managers”. Thus, considering the relatively lower number of men compared to women who were enrolled in the Mugello Study [25], our purpose is to assess the relationship between physically demanding work exposure with late-life physical performances, disability, general health status, and quality of life in a representative sample of nonagenarian women living in the Mugello area. The hypothesis behind this work is that physical work exposure during midlife may represent a risk factor for disability in the oldest-old.

## 2. Materials and Methods

### 2.1. Study Population

The study participants were enrolled within the community-based Mugello Study, a large epidemiological study involving people aged 90 years and over living in a rural area (Mugello, Tuscany, Italy); 504 persons were enrolled representing the 69% of the nonagenarians living in the area [25].

The study protocol has been described in detail elsewhere [25]. Data were collected from January 2011 to March 2012 during a home/nursing home visit through objective examinations and questionnaires. In brief, relevant geriatric items such as sociodemographic, functional and cognitive status, medical history, and clinical characteristics of participants were assessed. Moreover, participants underwent instrumental examinations and were administered several validated questionnaires assessing physical activity, mood, perceived health status, and quality of life. The study was conducted according to the Helsinki Declaration on Clinical Research Involving Human Subjects and was approved by the Don Carlo Gnocchi Foundation Ethics Committee. Informed written consent was obtained from all the participants, or their proxies, before their inclusion in the study. Inclusion criteria for this analysis were: female gender and having provided information about occupational history. Consistently to previous reports, based on differences between men and women in the work-related perception of demand and related health consequences [15,19,22,23,24], only the larger gender sample, represented by women, as commonly in extreme older, was included. Exclusion criteria were: the presence of severe blindness or deafness and the presence of severe dementia that would hamper the ability to perform a physical test [26]. Severe dementia was defined by a Mini-Mental State Examination (MMSE) score below 10 following indications provided by AIFA (Italian Medicines Agency), note 85 (http://www.agenziafarmaco.gov.it/it/content/nota-85, accessed on 24 March 2022).

### 2.2. Sociodemographic and Lifestyle

Data including age, gender, education, institutionalization, marital status, and smoke habits were collected by interview. Participants were subdivided into two groups according to the number of pregnancies; a cut-off of two pregnancies was chosen to obtain the evenest distribution of subjects. To assess the level of regular physical activity performed over the previous 12 months a questionnaire, modeled on the Harvard Alumni Questionnaire [27] and specifically adapted for Italian persons [28], was used. The questionnaire score ranges from 0 (sedentary) to 4 (intense physical activity several times a week). According to the score obtained, participants were divided into sedentary (no physical activity reported) and active (light to intense physical activity reported).

### 2.3. Medical History and Drug Consumption

Medical history was retrieved by interview and revision of medical records. The Charlson Comorbidity Index (CCI) was used to evaluate the cumulative burden of medical comorbidity [29]. The CCI includes 19 diseases and ranges from 0 to 33, with higher numbers representing a greater comorbidity burden. Participants were categorized into three groups according to cut-offs reported in the literature (CCI < 5, CCI between 6 and 8, CCI > 8) [30]. Information about the number of drugs taken was collected through interviews with the participants and by review of chronic drug prescriptions. According to cut-offs established in previous literature [31] participants were divided into three groups consistently with the number of drugs taken (0; 1–3; ≥4). Due to the small number of subjects belonging to the first group (no drugs taken, *n* = 12) the first two groups (0 and 1–3) were merged.

### 2.4. Physical and Functional Status

The Short Physical Performance Battery (SPPB) was administered to assess the lower extremities’ physical performance status [32]. Walking speed, standing balance, and the ability to stand up from a chair was evaluated. As a score lower than 10 has been used in literature to indicate mobility limitation and frailty [33], participants were divided into two groups according to the score obtained. Isometric handgrip strength was measured using a hydraulic dynamometer (RO+TEN, Verano Brianza, Italy). Grip strength was assessed in both hands and the highest of two right and left measurements was retained for analysis.

Functional independence was evaluated using the Katz questionnaire on Basic Activities of Daily Living (BADL), namely eating, bathing, dressing, toileting, and transferring [34]. According to the recommendation from the literature, suggesting that continence should be regarded as a separate dimension and difficulties in the bladder and/or bowel control should be considered as an impairment rather than a disability, continence was not considered [35]. Functional independence in performing Instrumental Activities of Daily Living (IADL) was assessed using the Lawton and Brody scale [36]. IADL evaluated were using the telephone, doing shopping, preparing meals, doing housework, doing laundry, traveling, taking medicine, and managing finances. Both concerning BADL and IADL, participants were defined as “not dependent” (equivalent to the wording “no BADL/IADL lost”) if they were independent in all items, otherwise the number of activities for which they needed assistance was recorded (BADL/IADL lost).

### 2.5. Cognitive and Psychological Status, Self-Perceived Quality of Life

The MMSE was used to evaluate the cognitive status [37]. The scale score ranges from 0 to 30 with lower values corresponding to a more compromised cognitive status. Raw MMSE scores were used, as score correction by age and education is not available for people aged 90 years and more. The possible presence of depressive symptoms was evaluated using a shorter version of the 15-item Geriatric Depression Scale [38]. Scores ≥8 were used to identify participants with moderate to severe depression. Health-related quality of life was evaluated using the 12-item Short-Form Health Survey (SF12) [39] which represents a more synthetic version of the 36-items Short-Form Health Survey [40]. The SF-12 assesses eight areas namely physical functioning, role physical, bodily pain, general health, vitality, social functioning, role emotional, and mental health. The raw scores of each item are coded, weighted, and summed to obtain the Physical Component Summary (PCS) and the Mental Component Summary (MCS). The scores range from 0 to 100, with higher scores indicating better quality of life. 

A detailed summary of scales and questionnaires used in the study is provided in the Supplementary Materials (Table S1).

### 2.6. Lifetime Occupation

Principal lifetime occupation was classified into six categories following the classification proposed by Geroldi and colleagues [14]: Group 1, white-collar workers (managers, executives, teachers, professionals); Group 2, tradesmen (shopkeepers) and craftsmen; Group 3, blue-collar workers (skilled and unskilled blue-collar, domestic service employees); Group 4, farmers; Group 5, housewives; Group 6, other employees (nurses, policemen, drivers, other occupations).

In this study the following occupations were included in the above-mentioned groups:

Group 1: teachers, managers, accountants, clerks

Group 2: merchants, hairdressers, butchers, chefs, shop assistants, tailors, embroiderers, barmaids, pharmacists

Group 3: blue-collars, domestic service employees, kitchen-maids, millers

Group 4: farmers

Group 5: women reporting no occupation other than housewife

Group 6: nurses, midwives

In addition, considering the historical period in which the participants lived, and their rural background, it was assumed that domestic work was done by women, even if involved in work activities. Consequentially, Group 5 (women declaring no other occupation than housewives) was considered as the reference group in this study, assumed to be the less physically demanding work, whereas Group 4 was assumed to be the main occupational demanding job.

### 2.7. Statistical Analysis

All statistical analyses were performed using SPSS 27.0 (SPSS Inc., Chicago, IL, USA). Data were firstly tested for normality using the Shapiro–Wilk test. Since data were not normally distributed (*p* < 0.05) continuous variables were summarized with their median value and interquartile range (IQR). Categorical and dichotomous variables were summarized through their median value and IQR, and through frequencies and percentages, respectively. Continuous sociodemographic, clinical, functional, and cognitive variables were compared between occupational categories using the Kruskal–Wallis test. Post-hoc analyses were performed using the Mann–Whitney U test with Bonferroni adjustment for type I errors of multiple comparisons. Categorical and dichotomous variables were compared among occupational groups using a Chi-Square test or Fisher’s exact test as appropriate according to the frequencies of variables. The association between functional parameters and lifetime occupation was investigated using multivariate regression analysis (logistic or linear as appropriate) where occupational categories represented the independent variable. Functional parameters that resulted to be significantly different between occupational groups were entered as the dependent variable. Any sociodemographic, clinical, functional, or cognitive variable that resulted significantly different between occupational categories was included in the analysis as a confounding factor. In all the above-mentioned analyses *p* < 0.05 was considered to indicate statistical significance.

## 3. Results

**Figure 1:**
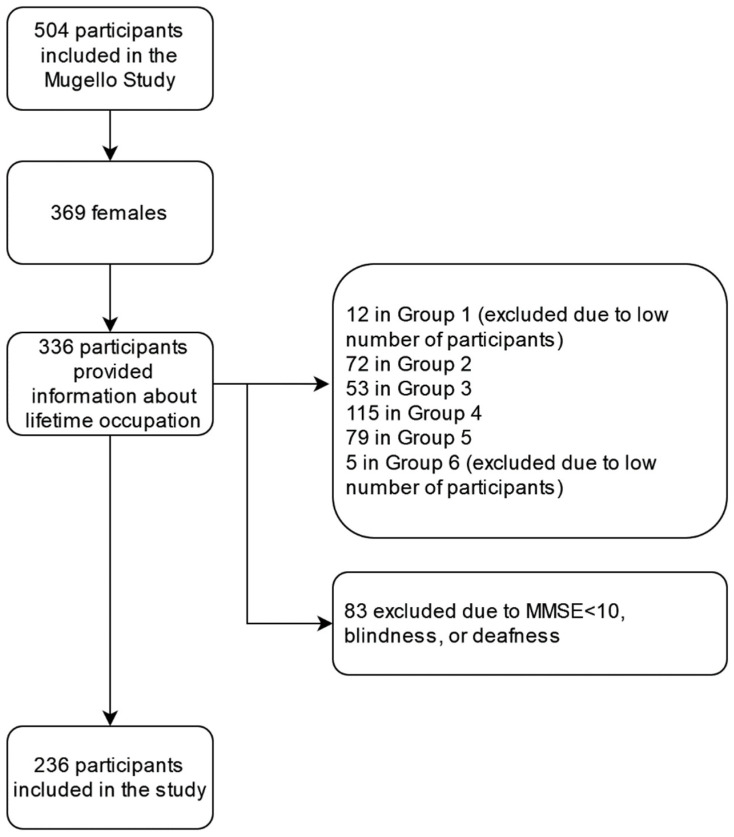
Flow diagram of the study participants.

Information about lifetime occupation was retrieved from 336 women participants of the Mugello Study. Participants belonging to White collars (*n* = 12) and other occupations were too few (*n* = 5) and were thus excluded from further analyses. In addition, 83 more subjects were excluded due to a low MMSE score (<10, *n* = 72), presence of deafness (*n* = 7), or blindness (*n* = 4). Two hundred thirty-six women were thus included in the study (Figure 1).

The general characteristics of the sample and participants belonging to different occupational groups are summarized in Table 1. The median age of the sample was 92 years (IQR: 4 years) with no significant differences between occupational groups (*p* = 0.754). Housewives reported a significantly longer work duration (median value 70 years, *p* < 0.001) but no significant differences were found between other occupations. A significant difference was found in education (*p* < 0.001) which was higher in Group 5 and Group 2 (median value 5 years) compared to Group 3 and Group 4 (median value 3 years). Participants were mainly non-institutionalized (216, 91.5%), widowed (221, 93.6%), and no smokers (201, 85.9%), with no significant differences among occupational groups. In Group 4, there was a significantly higher percentage of participants who had two or more pregnancies, compared to Group 2 and 3 (*p* < 0.001). A significantly higher MMSE (*p* = 0.001) was found in Group 2 (median value 25, IQR 6) compared to Group 4 (median value 21, IQR 9). Most of the participants had a CCI ≤ 5 (90, 38.3%) and used four or more medications (145, 61.7%) with no significant differences among the occupational groups. No significant difference (*p* = 0.639) was found in the mental component of the SF-12 questionnaire (Table 1), the median score reported for the entire sample was 46 (IQR 11). Similarly, no significant difference (*p* = 0.391) emerged concerning the mood, with 28.2% of the entire sample showing moderate to severe depression.

The functional characteristics of the sample group are summarized in Table 2. The median SPPB score was 3 (IQR 5) and most participants were classified as inactive (126, 53.8%). The median maximum handgrip measured was 12 kg (IQR 6). Median BADL and IADL lost in the entire group were one and three, respectively. The median physical component of the SF-12 questionnaire was 42 (IQR 12). None of the above-mentioned variables were significantly different among occupational groups. A significant difference between Group 4 and all other groups was found in the number of participants without limitations in performing BADL (no BADL lost). Group 4 had a fewer number of participants still independent in BADL (18, 20%) compared to other groups (Group 2: 39.6%, Group 3: 39.0%, Group 5: 40.4%; *p* = 0.020).

Being still independent in performing BADL was then entered in a logistic regression model as the dependent variable including occupational groups as the independent variable and MMSE score, education, and the number of pregnancies as confounders. Belonging to Group 4 was no longer associated with a reduced likelihood of being independent (*p* = 0.138, Table 3). On the contrary, having a higher MMSE score was significantly associated with independence in BADL (OR: 1.186, *p* < 0.001).

## 4. Discussion

In this study, we assessed the relationship between physically demanding work performed in middle age and late-life physical performance, functional independence, and quality of life in a large cohort of nonagenarian women living in an Italian rural area. Participants were for the most part farmers, followed by housewives, and blue-collar/domestic service employees. A very low number of participants were white-collar workers, which is coherent with what was observed in different sample groups of similar age [41].

As shown in Table 1 the median education was in the range of 3–5 years, in line with the historical period and similar to that reported in the EU-funded GEHA project conducted in oldest-old living in three Italian geographic areas [42]. As in Geroldi, a lower educational level in Group 4 (farmers) compared to the other worker categories was detected [14] and according to its educational level, Group 2 (trades-craftsmen) showed better cognitive performances compared to Group 4 (farmers). Differences in the occupational timeframe, superior for Group 5 compared to the others, were linked to the historical period (beginning of the twentieth century) when women started to work early as “housewives” and continued over time their activities. No differences across occupational groups were detected in physical performances assessed with subjective (questionnaire) and objective (SPPB, Handgrip max) measures. Results are in line with those obtained by McCarthy [9], where the association between functional limitation and physically demanding work was not present in the women sample. While it has been reported that manual workers are more depressed and with poorer health-related quality of life compared to other workers [16,17], in our sample, no differences were found among occupational groups.

The assessment of functional abilities revealed that Group 4 (farmers) had the lower number of subjects independent in the BADL compared to all the other works categories, supporting a higher risk of disability in association with a physically demanding job, as previously documented [9,10,11,12].

However, results obtained from the multivariate analysis reveal that physically demanding occupations in middle-aged exert an influence on the functional activity of nonagenarians, which disappears when cognition, one of the main determinants of disability, is considered. In our sample, differences in functional activity cannot be explained by a reduction in physical performances that were similar in all occupational groups. Thus, our data confirm the relevance of cognition in maintaining functional ability in older age [3,5,6,43] and are similar to that obtained by Geroldi in a younger population [14]. In our sample of very old women, cognition plays a relevant role in maintaining functional independence, supporting the need to promote intervention as geriatric rehabilitation and integrative treatment approaches using a multi-professional team setting to prevent or delay cognitive decline for maintaining participation in social life, self-care, and everyday skills. In this regard, recent evidence coming from the Finnish Geriatric Intervention Study to Prevent Cognitive Impairment and Disability (FINGER) study confirms the effectiveness of a multidomain intervention (inclusive of cognitive training, nutritional guidance, physical exercise, and management of metabolic and vascular risk factors) in reducing cognitive impairment and other chronic diseases development in the elderly [44,45].

Based on the results obtained, our hypothesis of an existing association between physically demanding work exposure and disability in the oldest-old could not be confirmed. This association was repeatedly observed in younger cohorts [9,10,11,12], suggesting the presence of a “short-term effect” of demanding work on disability, that tends to die out over time. Thus, seems appropriate to prevent work-related disability by applying interventions in a middle-aged employed population targeting both work-related conditions (reducing psychological stress and physical demand) and personal lifestyle factors (enhancing LTPA) [10,46,47].

A decrease in health disparity depending on the occupational status may be hypothesized in nonagenarians based on a “weakening effects” of working conditions after retirement, a “mortality selection” of people belonging to higher risk categories, and a “ceiling effect”, referring to a high risk of morbidity in all occupational categories. Regarding the “mortality selection” issue, indeed, it is worth mentioning that low skilled manual occupations are often considered as a proxy for a low socioeconomic status [48], a condition generally associated with a greater risk of poor health, higher rates of illness, disability, and mortality compared with belonging to high socioeconomic status [49].

### Limitations

Data presented in the study are collected 10 years ago; this represents a weakness of the study. However, since only in the last decades, changes have been made both in terms of improving workers safety and well-being, we believe that the conclusion of our study is valid even today. Moreover, to the best of the authors’ knowledge, the Mugello Study still represents one of the largest surveys conducted on the Italian population aged 90 years and over, and we believe that useful information may be extracted from its data, referring to a previous period. The study was conducted in a pre-COVID era, reflecting in part different health priorities and approaches compared to the actual ones; however, it is unlikely that the Covid pandemic may affect the (lack of) prospective association between midlife work activity and disability at a very old age. Among the limitations of this study is that we chose to perform our analysis only on women, thus our results cannot be generalized to the whole population of the oldest-old. Our choice was based on the low number of men compared to women who were enrolled in the Mugello Study (135 men, of which 108 were eligible) mainly farmers (40%) which prevented conducting the analysis also in the men subgroup. Levels of physical demanding work were not directly measured but attributed based on the job information reported by the participants, that is a limitation given by the retrospective nature of the survey which is worth acknowledging. Another limitation is that information on financial dissatisfaction was not available and only four of six occupational groups were numerically representative to be included in the analysis. Finally, since LTPA was performed by only 12 out of 236 participants, the variable did not enter the analysis. 

## 5. Conclusions

Few studies on the oldest old investigate the impact of exposures in working life on general health and disability. 

While confirming the well-known association between cognitive and functional decline in very old age, our results do not support the hypothesis that the negative effects of physical work exposure observed in midlife are relevant to predict disability in nonagenarian women.

## Figures and Tables

**Table 1:** Sociodemographic characteristics and general health of all participants and stratified by occupational group.

	*n*	Tot	Group 2 (*n* = 57)	Group 3 (*n* = 41)	Group 4 (*n* = 90)	Group 5 (*n* = 48)	*p*-Value
Age (years)	236	92 [4]	92 [5]	92 [4]	92 [4]	91.5 [4]	0.754
**Work duration (years)**	**227**	**47 [38]**	35 [40] *	30 [37] *	46 [39] *	**70 [15]**	**<0.001**
**Education (years)**	**234**	**3 [2]**	**5 [2] ^†,‡^** **range 1–11**	**3 [2]** **range 0–8**	**3 [2]** **range 0–5**	**5 [2]** **^†^** **range 0–8**	**<0.001**
Non-institutionalized	236	216 (91.5%)	50 (87.7%)	36 (87.8%)	85 (94.4%)	45 (93.8%)	0.378
BMI (kg/m^2^)	227	25 [6]	25 [7]	25 [7]	26 [5]	25 [7]	0.583
Marital status	236						0.342
Single		8 (3.4%)	4 (7%)	2 (4.9%)	1 (1.1%)	1 (2.1%)	
Married		7 (3.0%)	2 (3.5%)	1 (2.4%)	4 (4.4%)	0 (0%)	
Widowed		221 (93.6%)	51 (89.5%)	38 (92.7%)	85 (94.4%)	47 (97.9%)	
Use of tobacco	234						0.380
No		201 (85.9%)	44 (80%)	33 (80.5%)	82 (91.1%)	42 (87.5%)	
Previous history		29 (12.4%)	10 (18.2%)	7 (17.1%)	7 (7.8%)	5 (10.4%)	
Yes		4 (1.7%)	1 (1.8%)	1 (2.4%)	1 (1.1%)	1 (2.1%)	
**Pregnancies** **≥ 2**	**234**	**161 (68.8%)**	**27 (48.2%)** **^†^**	**21 (51.2%)** **^†^**	**79 (88.8%)**	**34 (70.8%)**	**<0.001**
**MMSE**	**236**	**23 [9]**	**25 [6]** **^†^**	**22 [10]**	**21 [9]**	**24.5 [11]**	**0.001**
CCI	236						0.397
≤5		123 (52.1%)	33 (57.9%)	16 (39.0%)	48 (53.3%)	26 (54.2%)	
6–8		92 (39.0%)	17 (29.8%)	22 (53.3%)	35 (38.9%)	18 (37.5%)	
>8		21 (8.9%)	7 (12.3%)	3 (7.3%)	7 (7.8%)	4 (8.3%)	
Number of drugs	235						0.403
<4		90 (38.3%)	27 (47.4%)	13 (31.7%)	33 (36.7%)	17 (36.2%)	
≥4		145 (61.7%)	30 (52.6%)	28 (68.3%)	57 (63.3%)	30 (63.8%)	
MCS	220	46 [11]	45 [11]	45 [8]	47 [11]	48 [11]	0.639
GDS > 8	227	64 (28.2%)	17 (30.9%)	7 (17.5%)	27 (31.8%)	13 (27.7%)	0.391

* Statistically different (*p* < 0.05) from Group 5. ^†^ Statistically different (*p* < 0.05) from Group 4. ^‡^ Statistically different (*p* < 0.05) from Group 3. Median (interquartile range) or *n* (%). BMI: Body Mass Index; MMSE: Mini-Mental State Examination; CCI: Charlson Comorbidity Index; MCS: Mental Component Summary; GDS: Geriatric Depression Scale.

**Table 2:** Functional characteristics of all participants and stratified by occupational group.

	*n*	Tot	Group 2 (*n* = 57)	Group 3 (*n* = 41)	Group 4 (*n* = 90)	Group 5 (*n* = 48)	*p*-Value
SPPB	235	3 [5]	4 [6]	2 [5]	2 [4]	3 [5]	0.511
Physical activity	234						0.137
Active		108 (46.2%)	27 (48.2%)	14 (34.1%)	39 (43.8%)	28 (58.3%)	
Sedentary		126 (53.8%)	29 (51.8%)	27 (65.9%)	50 (56.2%)	20 (41.7%)	
Handgrip max	224	12 [6]	12 [5]	12 [5]	12 [6]	15 [8]	0.335
BADL lost	231	1 [3]	1 [3]	1 [3]	1 [2]	1 [2]	0.067
**No BADL lost**	**231**	**74 (32.0%)**	**21 (39.6%)** **^†^**	**16 (39.0%)** **^†^**	**18 (20.0%)**	**19 (40.4%)** **^†^**	**0.020**
IADL lost	231	3 [6]	2 [7]	3 [6]	3 [7]	3 [5]	0.395
No IADL lost	231	49 (21.2%)	17 (32.1%)	7 (17.1%)	17 (18.9%)	8 (17.0%)	0.176
PCS	220	42 [12]	44 [14]	43 [11]	41 [11]	42 [12]	0.405

^†^ Statistically different (*p* < 0.05) from Group 4. Median [interquartile range] or *n* (%). SPPB: Short Physical Performance Battery; BADL: Basic Activities of Daily Living; IADL: Instrumental Activities of Daily Living; PCS: Physical Component Summary.

**Table 3:** Logistic regression analysis; association between occupational group and functional independence (no Basic Activities of Daily Living lost).

	B	S.E.	*p*-Value	OR	95%CI Low	95%CI High
Education (years)	−0.007	0.103	0.942	0.993	0.811	1.215
**MMSE**	0.171	0.035	0.000	1.186	1.106	1.271
Group 2 (Group 5 = ref)	−0.348	0.457	0.447	0.706	0.288	1.731
Group 3 (Group 5 = ref)	0.000	0.494	1.000	1.000	0.379	2.635
Group 4 (Group 5 = ref)	−0.672	0.453	0.138	0.511	0.210	1.241
Number of pregnancies ≥ 2	−0.299	0.358	0.403	0.741	0.368	1.494

Dependent variable: No Basic Activities of Daily Living lost. Nagelkerke R^2^: 0.224. MMSE: Mini-Mental State Examination.

## Data Availability

The data that support the findings of this study are available on request from the corresponding author [S.P.].

## Supplementary Materials

The following supporting information can be downloaded at: www.mdpi.com/article/10.3390/ijerph19148880/s1. Table S1: Surveys and scales used in the study.
