# Supplementary material for: Assessing Relationships between Physically Demanding Work and Late-Life Disability in Italian Nonagenarian Women Living in a Rural Area
Source: Int J Environ Res Public Health. 2022 Jul 21;19(14):8880. doi: 10.3390/ijerph19148880 (PMC9319548; doi:10.3390/ijerph19148880)
Supplement: Supplementary file 1 [file ijerph-19-08880-s001.zip › ijerph-1752913-supplementary.pdf]

International Journal of
Environmental Research and Public Health

MDPI

Supplementary Materials

Table S1. Surveys and scales used in the study.

Scale/test	Variable evaluated	Evaluation procedure	Range and interpretation	Reference
Harvard Alumni Questionnaire	Level of regular physical activity performed over the previous 12 months.	Administered through a questionnaire.	0 (sedentary) to 4 (intense physical activity several times a week)	[27]
Charlson Comorbidity Index (CCI)	Cumulative burden of medical comorbidity.	Calculated based on the presence of 19 conditions, each of which is assigned a weighted score of 1, 2, 3, or 6.	0-33<br>Higher scores indicate more coexisting conditions and a higher risk of death.	[29]
Short Physical Performance Battery (SPPB)	Lower extremities' physical performance status.	The SPPB test includes balance, gait speed, and chair stand tests. In the balance test, the participant stands with his/her feet together side by side, then with the side of the heel of one foot touching the big toe of the other foot, and finally with the heel of one foot<br>in front of and touching the toes of the other foot. Each exercise is performed for about 10 seconds.<br>The second test measures the 4 meters gait speed. The participant can use a cane and has two tries. In the third test, the participant stands up straight as quickly as he/she can five times without stopping in between, keeping the arms folded across the chest.	0 (worst performance) to 12 (best performance).	[32]
Katz questionnaire	Functional independence in performing Basic Activities of Daily Living (BADL).	Administered through a questionnaire. The Index ranks adequacy of performance in six functions: bathing, dressing, toileting, transferring, continence, and feeding.	0 (low function, dependent) to 6 (high function, independent)	[34]
Lawton and Brody scale	Functional independence in performing Instrumental Activities of Daily Living (IADL).	Administered through a questionnaire. The Index ranks adequacy of performance in eight functions: using the telephone, doing shopping, preparing meals, doing housework, doing laundry,	0 (low function, dependent) to 8 (high function, independent)	[36]

		traveling, taking medicine, and managing finances.		
Mini-mental state examination (MMSE)	Presence of cognitive impairment.	Administered through a 30- items questionnaire.	0 (severely compromised cognitive status) to 30 (cognitive status not compromised)	[37]
Geriatric Depression Scale (GDS)	Presence of symptoms of depression.	Administered through a 15-items questionnaire.	0 (no depressive symptoms) to 15 (severe depressive symptoms)	[38]
12-item Short-Form Health Survey (SF12)	Self-reported health-related quality of life.	Administered through a 12-items questionnaire that measure eight health domains to assess physical and mental health.	The total score ranges from 0 (poor quality of life) to 100 (great quality of life).	[39]
